# Application of machine learning to develop and validate a pain risk prediction model for patients with non-small cell lung cancer after video-assisted thoracoscopic surgery: A single-center retrospective study

**DOI:** 10.1097/MD.0000000000047025

**Published:** 2026-01-09

**Authors:** Feng Wang, Zhijie Qian, Jiawei Chen, Junjie Hu, Zhichao Wu

**Affiliations:** a Department of Thoracic Surgery, Changshu Hospital Affiliated to Nanjing University of Chinese Medicine, Changshu City, Jiangsu Suzhou, P.R. China.

**Keywords:** machine learning, non-small cell lung cancer, pain, predictive models, random forest, video-assisted thoracoscopic surgery

## Abstract

This study aimed to develop a machine learning (ML)-based model to identify risk factors for postoperative pain following video-assisted thoracoscopic surgery (VATS) lobectomy in non-small cell lung cancer (NSCLC) patients. This retrospective study analyzed data from 100 NSCLC patients who underwent VATS. Least absolute shrinkage and selection operator (LASSO) regression with 10-fold cross-validation identified predictive factors. Patients were split into training (80%) and testing (20%) sets. Seven ML algorithms were trained, with performance evaluated via receiver operating characteristic curve, sensitivity, specificity, and accuracy. The shapley additive explanations (SHAP) method interpreted the best-performing model. LASSO regression identified 11 predictors. The random forest (RF) model achieved the highest predictive performance (AUC: 0.901, 95% CI: 0.833–0.969). SHAP analysis highlighted elevated pro-gastrin releasing peptide, tumor volume, red cell distribution width, lactic dehydrogenase, and white blood cell count as risk factors, while dexmedetomidine and higher hemoglobin were protective. A simplified model retained comparable accuracy (DeLong test *P* = .4846). The RF-based ML model effectively predicts post-VATS pain risk in NSCLC patients, demonstrating potential to guide future research on preoperative risk assessment and personalized interventions. External validation in a larger cohort is required before clinical application.

## 1. Introduction

Lung cancer remains the leading cause of cancer-related mortality worldwide.^[1]^ From a pathological perspective, lung cancer is heterogeneous and non-small cell lung cancer (NSCLC) is a malignant tumor originating from the bronchial mucosa epithelium or alveolar epithelium, being the common pathological type of lung cancer.^[2]^ Video-assisted thoracoscopic surgery (VATS) is an emerging minimally invasive thoracic surgical technique first implemented in 1991,^[3]^ and the clinical use of VATS has increased significantly over the decades and is now the standard procedure for lung cancer. Compared with traditional thoracotomy, VATS has the advantages of less trauma, faster postoperative recovery, and better tolerance to adjuvant chemotherapy.^[4,5]^

Despite significant progress in the early diagnosis and treatment of chronic cancer pain, postoperative pain in lung cancer is still a common problem for patients and clinicians in the perioperative period.^[6]^ Despite the significant clinical advantages of VATS, a considerable number of patients still experience moderate to severe postoperative pain, which not only increases the psychological burden of patients, but also leads to the overuse of analgesic drugs and an increased risk of complications, prolongs the length of hospital stay and leads to an increase in medical costs.^[6,7]^ At present, the clinical management of postoperative pain still relies on opioid prescription drugs and adjuvant analgesics, and lacks personalized forecasting tools. Therefore, the development of potential tools to identify the development and intensity of postoperative pain is essential for the preventive management of post-VATS pain in NSCLC patients, and to improve the perioperative safety of patients. Affected by multiple factors such as age, gender, inflammatory parameters and other laboratory indicators, and surgery-related characteristics, postoperative pain degree showed significant individual differences.^[8–10]^ The establishment of an accurate postoperative pain prediction tool based on clinically readily available indicators will be conducive to the implementation of precision medicine, and is of great clinical significance for the elective management of surgery, the optimization of analgesic regimens and the improvement of patients’ prognosis. As of now, there are no prediction tools for predicting the risk of post-VATS pain in patients with NSCLC.

In order to improve the accuracy of clinical diagnosis or prediction, machine learning (ML) is gradually being used in the construction of clinical models.^[11]^ Thanks to the development of complex algorithm and the analysis of large datasets, ML can learn from data and make predictions or decision, which has been widely used in predicting the risk of complications and poor prognosis after cancer surgery. ML has shown a better prediction effect than traditional risk models such as logistic regression (LR), which can provide a reference for clinicians to formulate prevention and intervention plans.^[12]^ The purpose of this study was to screen the key predictors of post-VATS pain risk among NSCLC patients based on ML methods, and to construct a preliminary risk prediction tool, thereby providing a foundation for future research on the prevention and treatment of post-VATS pain in NSCLC patients.

## 2. Methods

### 2.1. Study design and participants

This retrospective study analyzed de-identified clinical data from 100 enrolled patients with pathologically confirmed NSCLC who underwent VATS at Changshu Affiliated Hospital of Nanjing University of Chinese Medicine between July 1, 2023 to July 1, 2024. The study protocol was approved by the Institutional Review Board of the hospital (Approval No.: CZYLS-2025051), which waived the requirement for written informed consent in accordance with national regulatory for retrospective studies using anonymized data. All procedures were conducted in compliance with the Declaration of Helsinki and relevant institutional guidelines.

Considering the potential for overfitting in ML models, we assessed the adequacy of our sample size using the events-per-variable (EPV). With 33 outcome events, a conservative rule of thumb of 5 EPV was applied. This yielded a minimum required sample size of 158 for the full-predictor model, indicating that our cohort of 100 patients was underpowered for this complex model and its results should be interpreted as exploratory. However, for the simplified 5-predictor model, the required sample size was 72, which was adequately met by our cohort (N = 100), providing statistical robustness to this simplified model which is the primary focus for clinical translation.

### 2.2. Data collection and quality control

Potential predictors of postoperative pain were systematically extracted from electronic medical records, encompassing demographic characteristics [age, gender, body mass index], preoperative comorbidities [hypertension, diabetes, dyslipidemia, cardiovascular disease (CVD)], laboratory parameters, and perioperative variables. Laboratory measures included coagulation markers [fibrinogen, procalcitonin, partial thromboplastin time, international normalized ratio, D-dimer], tumor biomarkers (pro-gastrin releasing peptide (ProGRP), neuron-specific enolase, carbohydrate antigen CA199), hepatic/renal function indices [alanine aminotransferase, aspartate aminotransferase, alkaline phosphatase, total bilirubin, albumin–globulin ratio, urea, creatinine, lactic dehydrogenase (LDH)], and hematological profiles [hemoglobin (HB), blood platelet count, mean arterial pressure, white blood cell count (WBC), lymphocyte count, red cell distribution width (RDW), mean platelet volume]. Perioperative data comprised American Society of Anesthesiologists physical status classification, intraoperative anesthetic dosages (fentanyl and dexmedetomidine in μg/kg), and surgical duration. All predictor variables were systematically collected within 24 hours preceding surgery to ensure temporal relevance for predictive modeling. Data acquisition was conducted under protocols approved by the Institutional review Board of Changshu Affiliated Hospital (Approval No.: CZYLS-2025051). All data were de-identified and stored on password-encrypted hospital servers, with strict confidentiality maintained throughout the study.

To minimize bias and ensure objectivity and accuracy in data collection, several rigorous measures were implemented during the data collection process. First, all involved medical staff received standardized training to ensure consistent and objective patient/family engagement and data entry. Second, regular quality checks were performed throughout the study to monitor data completeness and accuracy. Finally, an independent reviewer verified all collected data to ensure consistency and validity.

### 2.3. Definition of post-VATS pain

The outcome was early postoperative moderate-to-severe pain with 48 hours after VATS. The numeric rating scale is a one-dimensional assessment scale with a score of 0-10 points, with 0 being no pain and 10 being very painful. There are 4 levels of pain: no pain (0 point); mild pain (1–3 points); moderate pain (4–6 points), and severe pain (7–10 points).^[9,13]^ In the present study, patients with an numeric rating scale score of ≥4 within 48-hour after VATS were defined as having moderate to severe pain.

### 2.4. Feature selection using LASSO regression

The least absolute shrinkage and selection operator (LASSO) is a linear model that utilizes random sampling to construct a regression model and uses cross-validation to determine the optimal lambda value. LASSO regression controls the complexity of the model by penalizing project (a technique used to prevent model overfitting) and selects the most correlated predictive factors by compressing some regression coefficients to 0. In our study, LASSO regression was utilized to screen for predictive factors associated with post-VATS from candidate variables in the training set. To prevent data leakage, the entire dataset was first split into training (80%) and testing (20%) sets. The 10-fold cross-validation was used to calculate the mean square error of the LASSO regression model in different subsets, and the optimal LASSO regression parameters with the mean square error within the allowable range, and finally the predictive factors was screened.

### 2.5. Construction and validation of machine learning models

Seven representative ML algorithm models to predict postoperative pain after VATS were constructed based on the features selected by LASSO from the training set, including LR, random forest (RF), extreme gradient boosting, support vector machine, decision tree (DT), gradient boosting machines, and adaptive boosting. Model performance was assessed using the area under the receiver operating characteristic curve (AUC-ROC), sensitivity, specificity, accuracy, F1-score, positive predictive value (PPV), and negative predictive value.

To ensure robust evaluation of model generalizability and stability and to aid in model selection and hyperparameter tuning, we employed a repeated 5-fold cross-validation strategy on the training set.^[14]^ The average values of the evaluation indices from this cross-validation process were calculated to determine the model’s stability and performance during the development phase. The test set (20% of the total cohort) was used to provide the final, unbiased estimate of the model’s performance.

### 2.6. Model interpretability and clinical applicability assessment

The clinical translation of ML models is often hindered by their “Black-box” nature. To improve interpretability and evaluate clinical utility, we implemented a comprehensive validation framework combining multiple analytical approaches. This included ROC curves to quantify discriminative ability, decision curve analysis to assess net clinical benefit across decision thresholds, calibration plots to evaluate the alignment between predicted probabilities and observed outcomes, and Shapley additive explanations (SHAP) for quantifying feature contributions. Calibration curve is a graphical tool to assess the consistency between the predicted probability of a model and the observed results in practice. The SHAP framework was specifically applied to the best-performing model to interpret its predictions and identify the most influential predictors. Together, this integrated approach bridges statistical validation with clinical decision-making requirements by simultaneously evaluating model performance, calibration integrity, intervention utility, and interpretability.

### 2.7. Development and validation of a simplified prediction model

To improve clinical interpretability, we constructed a simplified prediction model using the top 5 most important features identified by the best-performing ML model. The predictive performance of this simplified model was statistically compared against the full feature set model using DeLong’s test for AUC comparison.

## 3. Statistical analysis

All statistical analyses and visualizations were performed using the R (v 4.4.4) programming language. Normally distributed continuous variables are presented as mean ± standard deviation (mean ± SD) and compared using the Student *t* test. Non-normally distributed continuous variables are summarized as median with interquartile range and compared using the Mann–Whitney *U* test. Categorical variables are expressed as frequency and percentage [n (%)] and compared between groups using the χ^2^ test or Fisher exact test, as appropriate. Two-sided *P* value < .05 was considered statistically significant.

## 4. Results

### 4.1. Characteristics of the study patients

A total of 100 patients with NSCLC undergoing VATS were included in this analysis, with 33 patients (33.0%) developing postoperative pain and 67 patients (67.0%) in the no-pain group. The cohort’s baseline characteristics were comprehensively detailed in Table 1.

**Table 1:** Baseline characteristics of NSCLC patients.

Variable	Total (n = 100)	Pain risk of post-VAST		Statistic	*P*
		No (n = 67)	Yes (n = 33)		
Age, M (Q_1_, Q_3_)	59.00 (53.00, 70.00)	59.00 (53.00, 69.50)	59.00 (53.00, 70.00)	*Z* = −0.242	.809
Gender, n (%)					
Male	33 (33)	23 (34.33)	10 (30.30)	χ^2^ = 0.162	.687
Female	67 (67)	44 (65.67)	23 (69.70)		
BMI, Mean ± SD	23.42 ± 3.56	23.41 ± 3.32	23.44 ± 4.06	*t* = −0.033	.974
Hypertension, n (%)					
No	61 (61)	44 (65.67)	17 (51.52)	χ^2^ = 1.863	.172
Yes	39 (39)	23 (34.33)	16 (48.48)		
Diabetes, n (%)					
No	88 (88)	59 (88.06)	29 (87.88)	χ^2^ = 0.000	1.000
Yes	12 (12)	8 (11.94)	4 (12.12)		
Dyslipidemia, n (%)					
No	97 (97)	67 (100.00)	30 (90.91)	–	.034
Yes	3 (3)	0 (0.00)	3 (9.09)		
Cardiovascular disease, n (%)					
No	97 (97)	67 (100.00)	30 (90.91)	–	.034
Yes	3 (3)	0 (0.00)	3 (9.09)		
ASA classification, n (%)					
1	51 (51)	38 (56.72)	13 (39.39)	χ^2^ = 2.655	.103
2	49 (49)	29 (43.28)	20 (60.61)		
MAP, Mean ± SD	103.88 ± 12.36	102.60 ± 12.51	106.48 ± 11.82	*t* = −1.488	.140
Greatest tumor diameter, cm, M (Q_1_, Q_3_)	1.20 (0.80, 1.80)	1.10 (0.75, 1.60)	1.50 (0.80, 2.20)	*Z* = −1.855	.064
Procalcitonin, M (Q_1_, Q_3_)	0.00 (0.00, 0.10)	0.00 (0.00, 0.10)	0.00 (0.00, 0.10)	*Z* = −0.183	.855
Fibrinogen, Mean ± SD	3.28 ± 0.70	3.32 ± 0.67	3.20 ± 0.77	*t* = 0.771	.443
Partial thromboplastin time, Mean ± SD	36.81 ± 3.75	36.99 ± 3.47	36.45 ± 4.30	*t* = 0.673	.503
International normalized ratio, M (Q_1_, Q_3_)	0.99 (0.95, 1.02)	0.99 (0.95, 1.02)	0.99 (0.95, 1.03)	*Z* = −0.393	.694
D-dimer, M (Q_1_, Q_3_)	0.18 (0.11, 0.28)	0.18 (0.10, 0.25)	0.18 (0.12, 0.31)	*Z* = −0.715	.474
ProGRP, M (Q_1_, Q_3_)	37.50 (28.35, 46.78)	36.10 (27.75, 43.35)	42.90 (29.20, 53.80)	*Z* = −1.789	.074
Nuronspecific enolase, M (Q_1_, Q_3_)	11.95 (10.20, 13.70)	12.10 (10.40, 13.75)	11.70 (10.10, 13.00)	*Z* = −0.960	.337
Carbohydrate antigen CA199, M (Q_1_, Q_3_)	7.83 (4.70, 12.32)	7.91 (4.40, 12.85)	7.66 (6.05, 10.40)	*Z* = −0.458	.647
Alanine aminotransferase, M (Q_1_, Q_3_)	18.00 (13.00, 25.25)	18.00 (13.50, 24.00)	20.00 (13.00, 26.00)	*Z* = −0.004	.997
Aspartate aminotransferase, M (Q_1_, Q_3_)	21.00 (18.00, 24.00)	21.00 (18.00, 23.50)	22.00 (17.00, 28.00)	*Z* = −0.834	.404
Alkaline phosphatase, Mean ± SD	85.78 ± 23.77	84.07 ± 21.48	89.24 ± 27.89	*t* = −1.023	.309
Total bilirubin, M (Q_1_, Q_3_)	14.40 (11.50, 18.00)	15.10 (11.75, 17.95)	13.00 (11.40, 18.60)	*Z* = −0.675	.500
Ratio of albumin to globulin, Mean ± SD	1.65 ± 0.27	1.63 ± 0.25	1.68 ± 0.31	*t* = −0.800	.426
Urea, M (Q_1_, Q_3_)	5.15 (4.30, 6.32)	5.60 (4.35, 6.30)	4.70 (4.00, 6.60)	*Z* = −1.540	.124
Creatinine, Mean ± SD	66.06 ± 14.12	65.69 ± 14.13	66.82 ± 14.27	*t* = −0.375	.708
Lactic dehydrogenase, Mean ± SD	187.75 ± 31.42	189.79 ± 30.51	183.61 ± 33.28	*t* = 0.925	.357
Hemoglobin, Mean ± SD	136.82 ± 14.92	137.45 ± 15.37	135.55 ± 14.09	*t* = 0.598	.551
White blood cell count, M (Q_1_, Q_3_)	5.20 (4.50, 5.93)	5.30 (4.50, 6.05)	5.20 (4.60, 5.80)	*Z* = −0.543	.587
Blood platelet count, M (Q_1_, Q_3_)	186.00 (154.75, 241.00)	187.00 (161.00, 255.00)	182.00 (141.00, 220.00)	*Z* = −1.228	.219
Lymphocyte, M (Q_1_, Q_3_)	1.60 (1.17, 2.00)	1.60 (1.30, 1.90)	1.60 (1.10, 2.10)	*Z* = −0.066	.947
Red cell distribution width, M (Q_1_, Q_3_)	12.70 (12.20, 13.12)	12.60 (12.10, 13.20)	12.70 (12.40, 13.10)	*Z* = −0.676	.499
Mean platelet volume, M (Q_1_, Q_3_)	11.35 (10.70, 12.33)	11.30 (10.70, 12.35)	11.50 (10.70, 12.30)	*Z* = −0.693	.488
Dose of fentanyl, µg, M (Q_1_, Q_3_)	70.00 (50.00, 80.00)	70.00 (50.00, 80.00)	70.00 (50.00, 80.00)	*Z* = −0.224	.823
Dose of dexmedetomidine, µg, M (Q_1_, Q_3_)	100.00 (100.00, 100.00)	100.00 (100.00, 100.00)	100.00 (100.00, 200.00)	*Z* = −2.189	.029
Duration of the operation, min, M (Q_1_, Q_3_)	95.00 (60.00, 141.25)	85.00 (60.00, 137.50)	110.00 (65.00, 145.00)	*Z* = −1.145	.252

χ^2^ = Chi-square test, – = Fisher exact, ASA = American Society of Anesthesiologists, BMI = body mass index, M = median, MAP = mean arterial pressure, NSCLC = non-small cell lung cancer, Q_1_ = 1st quartile, Q_3_ = 3rd quartile, SD = standard deviation, *t* = *t*-test, VATS = video-assisted thoracoscopic surgery, *Z* = Mann–Whitney test.

Key demographic and clinical variables were comparable between 2 groups. The median age was 59.00 years [interquartile range (IQR): 53.00–70.00] overall, with no significant difference between pain and no-pain groups (*P* = .809). Gender distribution (male: 33.00% overall) and body mass index (mean 23.42 ± 3.56) were also balanced (*P* > .05). Comorbidities including hypertension (39.00% overall) and diabetes (12.00% overall) showed no group differences (*P* > .05). Notably, dyslipidemia and cardiovascular disease prevalence significantly differed between groups (*P* = .034 for both). No patients with dyslipidemia (0/67) were observed in the no-pain group, whereas 9.09% (3/33) of the pain group had dyslipidemia. Similarly, cardiovascular disease was absent in the no-pain group but present in 9.09% (3/33) of the pain group. Perioperative variable revealed one significant predictor: patients developing pain received a higher median intraoperative dexmedetomidine dose [200.00 μg (IQR: 100.00–200.00) vs 100.00 μg (IQR: 100.00–100.00), *P* = .029]. Other laboratory markers (e.g., fibrinogen, D-dimer, tumor biomarkers) and operative duration [median 95.0 minutes, IQR: 60.00–141.25] showed no statistical differences between 2 groups (*P* > .05).

### 4.2. Identification of predictors using LASSO regression

To mitigate multicollinearity and reduce overfitting, we performed feature selection using LASSO regression. Through 10-fold cross-validation, the optimal penalty parameter (λ) was identified as 0.057. This process resulted in the selection of 11 best pain predictors were finally selected: ProGRP, dexmedetomidine, APTT, tumor size, LDH, RDW, WBC, HB, American Society of Anesthesiologists, CVD, and dyslipidemia.

### 4.3. Development and validation of the pain risk prediction model

**Figure 1.**
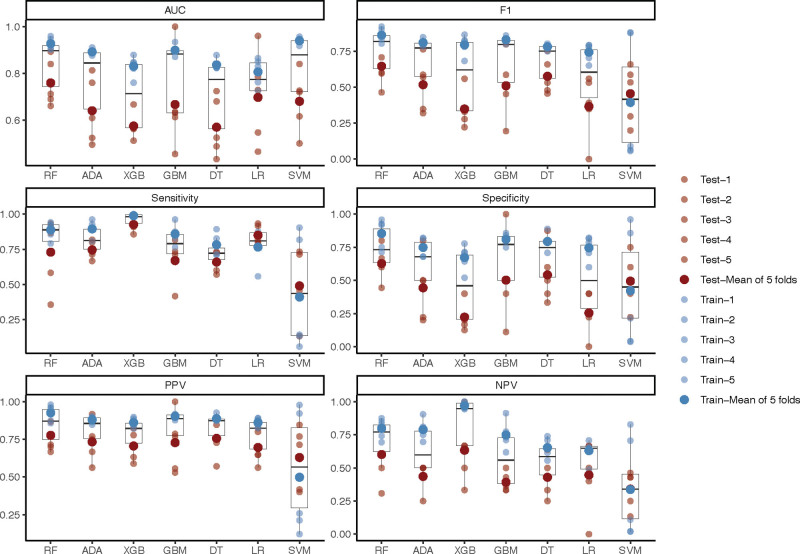
Distribution of performance metrics for 7 machine learning models in predicting post-VATS pain. Notes: Boxplots show the distribution of AUC, F1-score, sensitivity, specificity, PPV, and NPV for logistic regression (LR), random forest (RF), extreme gradient boosting (XGB), support vector machine (SVM), decision tree (DT), gradient boosting machines (GBM), and adaptive boosting (ADA). The plot provides an overview of model stability and comparative performance. AUC = area under the receiver operating characteristic curve, NPV = negative predictive value, PPV = positive predictive value.

We developed prediction models for moderate-to-severe pain after VATS in NSCLC patients using 7 ML algorithms: LR, RF, extreme gradient boosting, support vector machine, DT, gradient boosting machines, and adaptive boosting. The distribution of performance metrics (AUC, F1-score, sensitivity, specificity, PPV, and negative predictive value) across all cross-validation folds for each model were visualized in Figure 1, providing a comprehensive overview of their stability and comparative efficacy.

**Figure 2.**
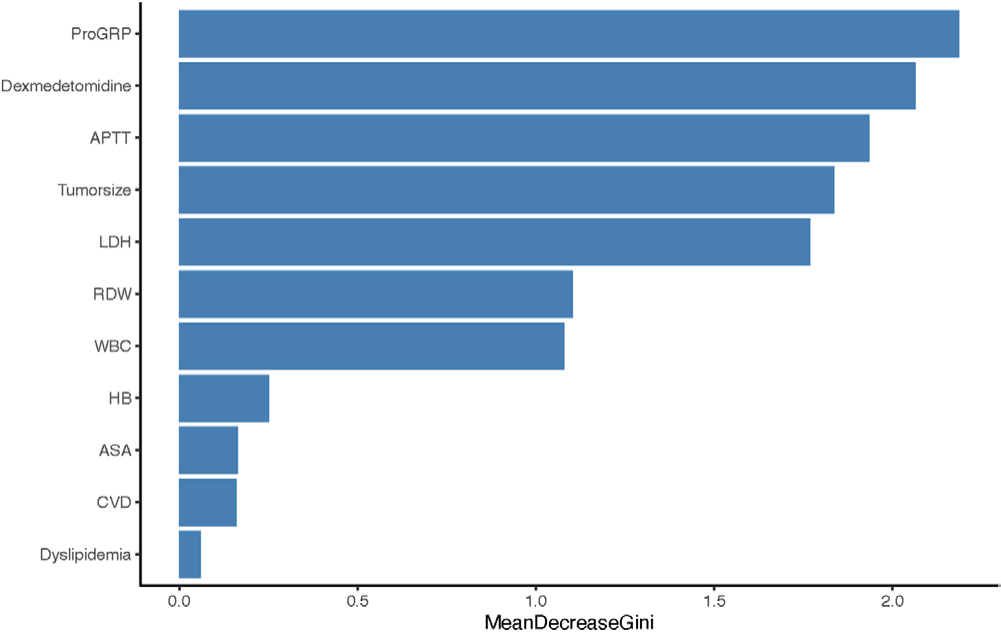
Feature importance of the top 11 predictors for post-VATS pain. Notes: The relative contribution of the top 11 predictor variables in forecasting moderate-to-severe pain after video-assisted thoracoscopic surgery (VATS) is displayed. The features are ranked in descending order of their importance as derived from the random forest model.

Based on this comparative analysis, the RF model consistently demonstrated superior performance. As summarized in Table 2, the RF model achieved the highest AUC of 0.927 (±0.025) during 5-fold cross-validation on the training set, indicating exceptional learning capability and stability. Most importantly, this strong performance was confirmed on the held-out test set, where the RF model attained an AUC of 0.893, along with the highest values in accuracy (0.900), sensitivity (0.933), and PPV (0.933) among all models evaluated. Therefore, the RF model was identified as the optimal predictor and selected for subsequent interpretability and simplification analyses. The mean decrease gini is an indicator used in the RF algorithm to assess the importance of a feature factor, which measures the contribution of a feature to the model’s classification ability based on Gini impurity. Figure 2 illustrated the contribution of 11 risk factors to predict the risk of moderate to severe pain. Among these pain predictor factors, ProGRP levels, dexmedetomidine use, APTT, tumor size, LDH levels, RDW levels, and WBC levels demonstrated higher predictive contributions for post-VATS pain risk among NSCLC patients.

**Table 2:** The predictive performance of the RF algorithm.

Set	AUC (95% CI)	Accuracy	Sensitivity	Specificity	PPV	NPV	F1-score
Training (5-fold CV Mean ± SD)	0.927 ± 0.025	0.875 ± 0.034	0.888 ± 0.063	0.854 ± 0.113	0.926 ± 0.056	0.799 ± 0.077	0.864 ± 0.045
Test (hold-out)	0.893 (0.704–1.000)	0.900	0.933	0.800	0.933	0.800	0.860

AUC = area under the receiver operating characteristic curve, CV = cross-validation, NPV = negative predictive value, PPV = positive predictive value, RF = random forest, SD = standard deviation.

* Note: Training set performance is presented as the mean ± standard deviation (SD) across the 5 cross-validation folds. The test set performance is from a single evaluation on the held-out dataset (20% of the total cohort, corresponding to Fold 1 Test set in the original analysis).

### 4.4. Model interpretability based on SHAP analysis

**Figure 3.**
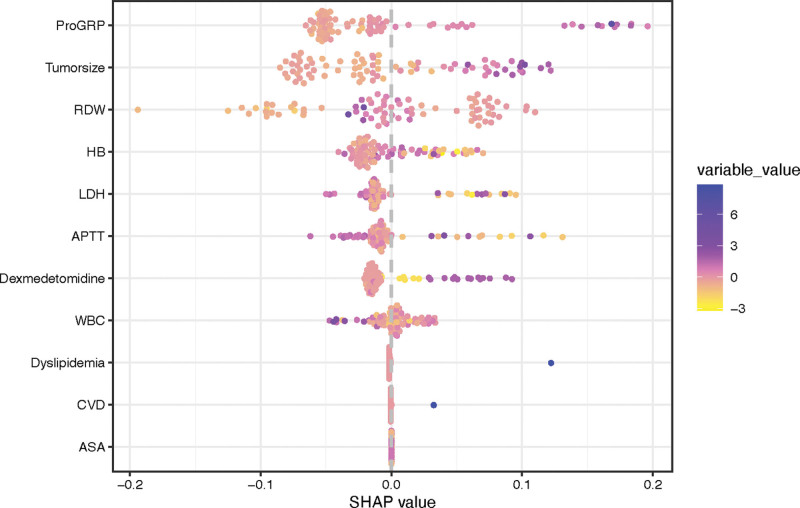
Interpretability of the optimal random forest (RF) model using Shapley additive explanations (SHAP) summary plot. Notes: This plot illustrates the impact of the top predictors on the model’s output for forecasting moderate-to-severe pain. Each point represents an individual patient. Features are ranked from top to bottom by their overall importance (mean absolute SHAP value). The color represents the feature’s actual value for that patient, from low (yellow) to high (blue).

Given its superior predictive performance, the RF model was selected for further interpretability analysis. Figure 3 provided a holistic visualization of predictor contributions using SHAP summary plot. Each point represents an individual patient, where color indicates the feature value: blue corresponds to higher values and yellow to lower values. The horizontal position of each point reflects the magnitude and direction of the feature’s impact on model output, with points farther from the SHAP value of 0 exerting greater influence on the prediction.

This way, a feature relationship with the SHAP value can be better understood. In these 11 predictive factors, the patients’ ProGRP, tumor size, RDW, HB, LDH, dexmedetomidine dose, ATPP and WBC played important role compared with the other predictive factors of which the distribution of SHAP values was largely clustered near the center.

### 4.5. Simplification of a simplified RF model

**Figure 4.**
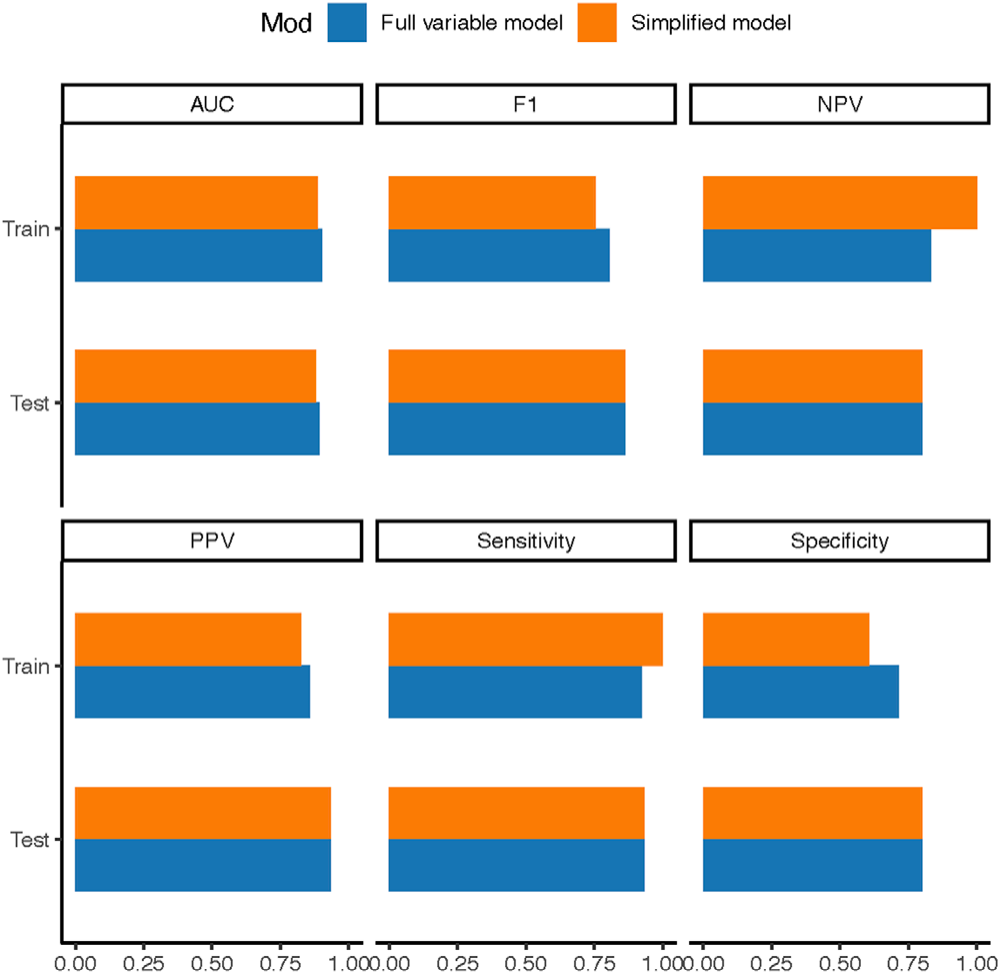
Robustness assessment of the simplified prediction model through cross-validation performance distribution. Notes: The distribution of key performance metrics across all cross-validation folds for the final simplified model is shown.

**Figure 5.**
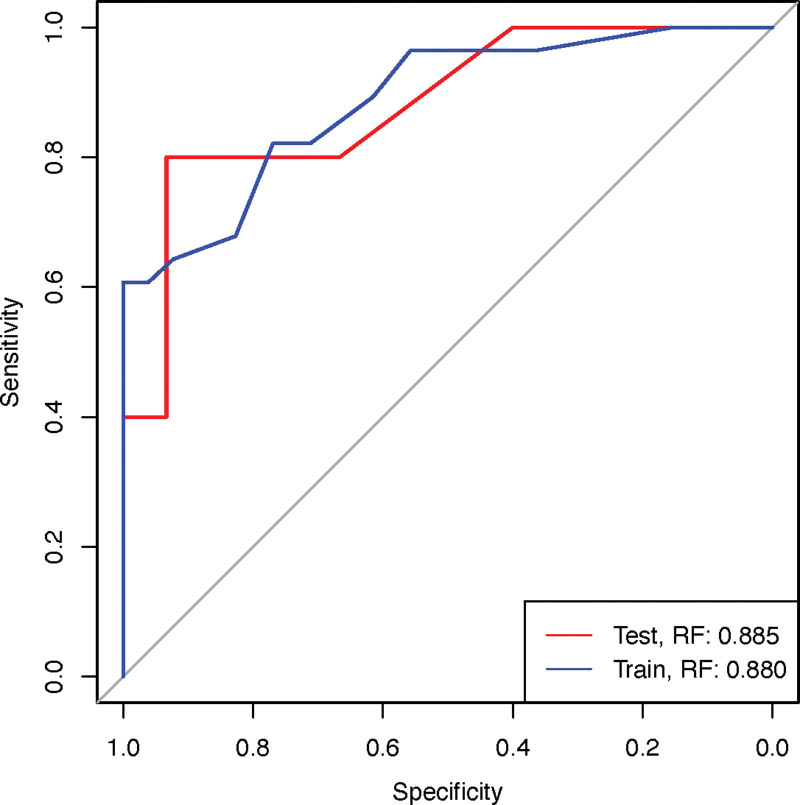
Receiver operating characteristic (ROC) curve of the simplified prediction model. Notes: The ROC curve evaluates the model’s ability to discriminate between patients with and without moderate-to-severe pain after VATS. VATS = video-assisted thoracoscopic surgery.

To improve clinical applicability, we constructed a simplified RF model using the top 5 most influential predictors identified in the original model. The performance metrics of this simplified model were summarized in Table 3. We employed DeLong test to compare the AUC-ROC values between the simplified and full models, which revealed no statistically significant difference (all *P* of Delong test > .05), indicating preserved discriminative ability despite feature reduction. It is noteworthy that while the performance of the simplified model was not statistically inferior, the AUC values were numerically lower than those of the full model (e.g., test set AUC: 0.880 vs 0.893). This trade-off between a marginal, non-significant decrease in predictive accuracy and a substantial gain in model simplicity and potential for clinical adoption is well-justified. The robustness of the simplified model was further supported by the performance distribution shown in Figure 4. Finally, the ROC curve of the simplified model was presented in Figure 5, demonstrating its maintained predictive capacity.

**Table 3:** The predictive performance of the simplified model.

Dataset	AUC	F1	Accuracy	Sensitivity	Specificity	PPV	NPV	Delong test
Train	0.885 (0.807–0.962)	0.76	0.863 (0.767–0.929)	1.000 (1.000–1.000)	0.607 (0.426–0.788)	0.825 (0.732–0.919)	1.000 (1.000–1.000)	.4846
Test	0.880 (0.690–1.000)	0.86	0.900 (0.683–0.988)	0.933 (0.807–1.000)	0.800 (0.449–1.000)	0.933 (0.807–1.000)	0.800 (0.449–1.000)	.7358

AUC = area under the curve, NPV = negative predictive value, PPV = positive predictive value.

## 5. Discussion

This retrospective study analyzed clinical data from 100 NSCLC patients who underwent VATS at the Department of Thoracic Surgery, Changshu Affiliated Hospital of Nanjing University of Chinese Medicine between July 2023 and May 2024. Seven ML-based predictive models for post-VATS pain risk were developed and evaluated. Comparative analysis revealed that the RF model demonstrated superior accuracy and stability. The RF algorithm has emerged as a powerful and widely used tool for both classification and regression tasks, enhancing predictive performance through the ensemble of multiple DTs with voting or averaging mechanisms.^[15]^ Numerous clinical studies have employed RF algorithms to construct predictive models for postoperative complications and prognosis in cancer patients. Our findings are consistent with previous studies employing RF algorithms for pain prediction. For instance, Li et al^[16]^ developed a robust predictive model for lung cancer-related pain using the RF algorithm, with both AUC values and calibration curves demonstrating high accuracy in predicting pain risk. Wakabayashi et al^[17]^ developed a predictive model for post-radiotherapy pain response in patients with painful spinal metastases receiving palliative treatment using RF ensemble learning. The RF models constructed based on radiomic features, clinical features, and “combined” features achieved AUC values of 0.824, 0.702, and 0.848, respectively. In this study, the prediction model constructed by the RF algorithm demonstrated superior predictive performance compared to the other 6 algorithm models. The AUC in the training set was 0.901 (95% CI: 0.833–0.969).

SHAP analysis revealed that post-VATS pain prediction in NSCLC patients was primarily driven by: tumor biomarkers (ProGRP), surgical trauma (tumor size), inflammatory/metabolic indicators (RDW, LDH, WBC), and anesthetic management (dexmedetomidine use). Gastrin-releasing peptide (GRP), a gastrointestinal hormone, primarily stimulates gastrin secretion from gastric G cells, modulates smooth muscle contraction, and facilitates cell-to-cell communication. As the precursor of GRP, ProGRP exhibits a longer half-life and greater stability.^[18]^ Elevated ProGRP expression correlates with enhanced inflammatory responses in the tumor microenvironment, potentially amplifying postoperative inflammatory reactions through the release of pro-inflammatory factors (IL-6, TNF-α, prostaglandin E2, etc), which may directly stimulate nociceptive nerve endings.^[19]^ Elevated RDW may indicate anemia or chronic inflammatory states. In cancer patients, chronic inflammation suppresses erythrocyte maturation, leading to abnormal red blood cell size heterogeneity (anisocytosis). Chronic anemia can induce tissue hypoxia, activating hypoxia-inducible factors that stimulate nociceptive nerve endings and exacerbate pain perception.^[18]^ LDH, a biomarker of cellular damage, is frequently elevated in tumor necrosis or metastatic progression.^[20]^ Increased WBC counts reflect systemic or localized inflammatory activity. The infiltration of inflammatory cells (neutrophils, monocytes, etc) into the tumor microenvironment promotes the release of algogenic mediators like prostaglandin E2 (PGE2) and bradykinin, which directly sensitize peripheral nociceptors.^[21]^ Dexmedetomidine is a highly selective α2-adrenergic receptor agonist with sedative, analgesic, and anxiolytic properties. It potentiates the analgesic effects of opioids while suppressing the release of pro-inflammatory cytokines, thereby attenuating postoperative or tumor-associated inflammatory responses and indirectly alleviating inflammatory pain.^[22]^

In recent years, ML has been widely applied in predicting post-operative pain risk in cancer patients. Its automated learning architecture facilitates better interpretation of pain-related variables, thereby assisting healthcare professionals in making more precise diagnoses and treatment decisions. This study integrated patient demographics, tumor characteristics, laboratory parameters, and comorbidities to develop a predictive model for post-VATS pain risk in NSCLC patients. The model enables preoperative identification of high-risk patients, allowing for personalized analgesic strategies such as optimized anesthetic selection (e.g., dexmedetomidine administration) or adjusted postoperative pain management protocols incorporating multimodal analgesia to reduce both the incidence and severity of postoperative pain. Furthermore, pain-related predictors identified through SHAP values and LASSO regression may help elucidate potential connections between pain mechanisms and tumor biology. With continued advancements in medical information technology, ML-based approaches can expand NSCLC-related research, promote precision medicine and individualized therapy, and ultimately contribute to more accurate and clinically practical predictive models.

However, this study has several limitations that should be considered when interpreting the results. First, the most significant constraint is the relatively small size and the limited number of outcome events, which, despite our use of LASSO regularization for feature selection and rigorous internal validation techniques, inherently increase the risk of model overfitting and may lead to optimistic performance estimates. The EPV analysis confirmed that our cohort was underpowered for the full 11-predictor model, reinforcing that its findings should be interpreted as exploratory. However, the simplified 5-predictor model, which is our primary candidate for clinical translation, met the sample size requirement (EPV > 10 for events), providing greater confidence in its robustness. Second, although we implemented rigorous methodological safeguards, we must acknowledge the theoretical risk of data leakage and its potential impact on model performance. Data leakage can occur when information from the test set inadvertently influences the model training process, leading to overly optimistic performance estimates. To mitigate this critical risk, the entire dataset was first split into training (80%) and testing (20%) sets before any feature selection or model development. The LASSO regression for feature selection was performed exclusively on the training set. The predictive features identified by LASSO were then fixed and applied to both the training and testing sets for model development and evaluation, respectively. While this stratified split and strict separation protocol significantly reduces the risk of data leakage, we acknowledge that in a small single-center sample, the variance in a single test set could still influence the final performance metrics. External validation is ultimately required to fully confirm the model’s generalizability. Other limitations include the retrospective, single-center design, which may affect the generalizability of our findings and introduces the potential for unmeasured confounding. The patient population, surgical techniques, and perioperative management protocols from a single institution may not be representative of other centers. Consequently, the performance of our prediction model and the specific predictor-outcome relationships identified require validation in larger, multi-center cohorts before they can be considered broadly applicable. Finally, while SHAP analysis was employed to enhance interpretability, the inherent “black-box” nature of complex ensemble models like RF continues to pose a challenge for full clinical transparency.

Given these limitations, our future research directions are clear. The essential next step is the external validation of this prediction model in large, multi-center, prospective cohort. Such validation is crucial to verify the model’s discriminative ability across diverse patient populations and clinical settings, to refine the predictor set, and ultimately, to assess its impact on clinical decision-making and patient outcomes in a real-world scenario.

## 6. Conclusion

This study successfully developed and validated a ML-based predictive model for post-VATS pain risk in NSCLC patients. Core predictive factors including ProGRP level, tumor size, dexmedetomidine usage, HB, RDW, and LDH were identified as pivotal determinants. This pilot study successfully developed an interpretable ML model that identifies key predictors for post-VATS pain. The model demonstrates significant potential for future development towards transforming postoperative pain management from empirical intervention to precision prevention, pending validation in larger cohorts.

## Author contributions

**Data curation:** Jiawei Chen.

**Formal analysis:** Zhijie Qian, Jiawei Chen, Junjie Hu.

**Investigation:** Zhijie Qian, Zhichao Wu.

**Methodology:** Feng Wang, Zhijie Qian, Zhichao Wu.

**Resources:** Junjie Hu, Zhichao Wu.

**Software:** Jiawei Chen, Zhichao Wu.

**Supervision:** Jiawei Chen, Zhichao Wu.

**Validation:** Zhichao Wu.

**Writing – original draft:** Feng Wang.
